# Use of multistate Bennett acceptance ratio method for free-energy calculations from enhanced sampling and free-energy perturbation

**DOI:** 10.1007/s12551-022-01030-9

**Published:** 2022-12-14

**Authors:** Yasuhiro Matsunaga, Motoshi Kamiya, Hiraku Oshima, Jaewoon Jung, Shingo Ito, Yuji Sugita

**Affiliations:** 1 Graduate School of Science and Engineering, Saitama University, Saitama, Saitama 338-8570 Japan; 2 Institute for Molecular Science, Myodaiji, Okazaki, Aichi 444-8585 Japan; 3 Laboratory for Biomolecular Function Simulation, RIKEN Center for Biosystems Dynamics Research, Kobe, Hyogo 650-0047 Japan; 4 Computational Biophysics Research Team, RIKEN Center for Computational Science, Kobe, Hyogo 650-0047 Japan; 5 Theoretical Molecular Science Laboratory, RIKEN Cluster for Pioneering Research, Wako, Saitama 351-0198 Japan

**Keywords:** Multistate Bennett acceptance ratio, Umbrella sampling, Replica exchange molecular dynamics, Free-energy perturbation, Molecular dynamics, Enhanced conformational sampling, Free-energy calculation, QM/MM calculations

## Abstract

Multistate Bennett acceptance ratio (MBAR) works as a method to analyze molecular dynamics (MD) simulation data after the simulations have been finished. It is widely used to estimate free-energy changes between different states and averaged properties at the states of interest. MBAR allows us to treat a wide range of states from those at different temperature/pressure to those with different model parameters. Due to the broad applicability, the MBAR equations are rather difficult to apply for free-energy calculations using different types of MD simulations including enhanced conformational sampling methods and free-energy perturbation. In this review, we first summarize the basic theory of the MBAR equations and categorize the representative usages into the following four: (i) perturbation, (ii) scaling, (iii) accumulation, and (iv) full potential energy. For each, we explain how to prepare input data using MD simulation trajectories for solving the MBAR equations. MBAR is also useful to estimate reliable free-energy differences using MD trajectories based on a semi-empirical quantum mechanics/molecular mechanics (QM/MM) model and ab initio QM/MM energy calculations on the MD snapshots. We also explain how to use the MBAR software in the GENESIS package, which we call *mbar_analysis*, for the four representative cases. The proposed estimations of free-energy changes and thermodynamic averages are effective and useful for various biomolecular systems.

## Introduction

Multistate Bennett acceptance method (MBAR) (Shirts and Chodera 2008) is widely used to estimate free energy changes between different states and thermodynamic averages at the states of interest using simulation trajectory data after the simulations have been finished. MBAR is an extension of Bennett’s acceptance ratio method (Bennett 1976) that was formulated for only two states to multiple states. Theoretically, the estimation using MBAR is superior to other estimators in that it has the lowest variance and is asymptotically unbiased (Shirts and Chodera 2008). MBAR can be interpreted as the limit of infinitesimal bin size in the weighted histogram analysis method (WHAM) (Kumar et al. 1992),(Souaille and Roux 2001),(Tan et al. 2012). It can avoid any biases introduced by the binning of trajectory data.

MBAR covers a broad range of applications where thermodynamic ensembles are required. They include the estimations of the free-energy differences including absolute/relative binding free-energy calculations (Chodera et al. 2011) and the ensemble averages including the potential of mean force (PMF) calculations from biased molecular dynamics (MD) trajectories, such as umbrella sampling (Torrie and Valleau 1977), or temperature replica-exchange MD (T-REMD) (Sugita and Okamoto 1999). Furthermore, MBAR has recently been applied to the rapid parameterization of all-atom force-field parameters (Messerly et al. 2018) and coarse-grained model parameters (Shinobu et al. 2019).

One of the practical difficulties in MBAR is to solve the nonlinear simultaneous equations numerically. This requires a sufficient memory capacity on the order of the square of the number of states multiplied by the number of samples. The computational complexity in solving the equations drastically increases with the number of states, which is a bottleneck in large-scale applications with MBAR. To solve this problem, Zhang et al. (2015) and Tan et al. (2016) proposed stochastic solvers, where locally weighted histogram analysis is used to approximate the global solution of the MBAR equations. Recently, Ding et al. developed a rapid solver inspired by the fact that the MBAR equations can be derived as a Rao-Blackwell estimator (Ding et al. 2019).

Another difficulty in using the MBAR equations is how to prepare the input data using different types of MD simulation trajectories. The MBAR equations require the potential energies and thermodynamic parameters of the system as input data. However, in many practical applications, it is often not necessary to use all the potential energy terms in the MBAR equations. For example, in the case of umbrella sampling (US), the potential energies of the system excluding the restraint energies are canceled out in the MBAR equations. The restraint energies alone are sufficient as input data. Another example is the case of MD simulations at constant volume, where $pV$ terms can be canceled out; thus, it is not necessary for solving the MBAR equations. Because it is difficult for non-experts to recognize which terms can be canceled out, users may attempt to prepare whole system’s potential energies by themselves. This leads to tedious efforts in preparing unnecessary input data and limits the uses of the MBAR by non-expert users.

In this review, we summarize the basic theory of the MBAR equations and describe representative usages for different types of MD simulation trajectories, such as enhanced conformational sampling methods like umbrella sampling (US) (Torrie and Valleau 1977), temperature replica-exchange MD (T-REMD) (Sugita and Okamoto 1999), and replica exchange with solute tempering (REST/REST2/gREST) (Liu et al. 2005) (Wang et al. 2011) (Terakawa et al. 2011) (Kamiya and Sugita 2018), and free-energy perturbation (FEP) (Zwanzig 1954) (Tembre and Mc Cammon 1984) (Mey et al. 2020). MBAR is also useful for estimating more reliable free-energy changes using MD simulations based on semi-empirical quantum mechanics/molecular mechanics (QM/MM) and ab initio QM/MM calculations (Warshel and Levitt 1976) on the MD snapshots (Yagi et al. 2021). As examples, we present several computational results using the MBAR equations for free-energy estimator for simple biological systems. Finally, we will discuss our perspectives on free-energy estimation methods.

## The classifications of MBAR applications

### The MBAR equations

First, we define ${\varvec{x}}\in\Gamma$ as the configurations of the target system to be simulated. Here, $\Gamma$ is the configuration space. We also define that ${\varvec{n}}\left({\varvec{x}}\right)$ is the number of molecules of each of *M* components of the system, and ${\varvec{\mu}}$ is the vector of chemical potentials of the corresponding components. In the MBAR formulation, state *i* is specified by a combination of potential energy function ${U}_{i}({\varvec{x}})$, inverse temperature ${\beta }_{i}$, pressure ${p}_{i}$, and/or chemical potential(s) ${{\varvec{\mu}}}_{i}$, or their conjugate variables depending on the ensemble. Here, the inverse temperature ${\beta }_{i}$, the pressure ${p}_{i}$, and/or the chemical potential(s) ${{\varvec{\mu}}}_{i}$ are given as external parameters in the input of MD simulation. For example, the inverse temperature ${\beta }_{i}$ corresponds to that of the thermostat. Suppose that we obtain ${N}_{i}$ uncorrelated samples in state *i* out of *K* states, and for each state *i*, ${U}_{i}({\varvec{x}})$, ${\beta }_{i}$, ${p}_{i}$, and ${{\varvec{\mu}}}_{i}$ are defined. Then, the reduced potential energy ${u}_{i}({\varvec{x}})$ of state *i* is then defined by Shirts and Chodera (2008):

1 $${u}_{i}\left({\varvec{x}}\right)={\beta }_{i}\left[{U}_{i}\left({\varvec{x}}\right)+{p}_{i}V\left({\varvec{x}}\right)+{{\varvec{\mu}}}_{i}^{T}{\varvec{n}}\left({\varvec{x}}\right)\right]$$

where ${\varvec{n}}({\varvec{x}})$ are the numbers of molecules corresponding to the chemical potentials.

The dimensionless free energy of state *i* can be defined using the integral of the Boltzmann weight:

2 $${f}_{i}=-\mathrm{ln}\int {e}^{-{u}_{i}\left({\varvec{x}}\right)}d{\varvec{x}}$$

Note that the above equations can use arbitrary weights other than the Boltzmann factor. The free energy difference between two states, $\Delta {f}_{ij}={f}_{i}-{f}_{j}$, is estimated using the MBAR equations as discussed below.

The MBAR equations give us the best estimator for free energy differences between different states from MD simulation data. Let ${\left\{{{\varvec{x}}}_{in}\right\}}_{n=1}^{{N}_{i}}$ be configurations sampled in state *i*, then the equations are given by:

3 $${\widehat{f}}_{i}=-\mathrm{ln}\sum_{j=1}^{K} \sum_{n=1}^{{N}_{j}} \frac{\mathrm{exp}\left[-{u}_{i}\left({{\varvec{x}}}_{jn}\right)\right]}{\sum_{k=1}^{K} {N}_{k}\mathrm{exp}\left[{\widehat{f}}_{k}-{u}_{k}\left({{\varvec{x}}}_{jn}\right)\right]}$$

Here, ${\widehat{f}}_{i}$ are the solutions to the MBAR equations. ${\widehat{f}}_{i}$ are determined up to an additive constant, so only their differences $\Delta {\widehat{f}}_{ij}={\widehat{f}}_{i}-{\widehat{f}}_{j}$ are meaningful. Since ${\widehat{f}}_{i}$ is found on the right-hand side of Eq. (3) as well as the left-hand side, the equation is solved by self-consistent iterations or the Newton–Raphson method until the convergence of ${\widehat{f}}_{j}$. On the other hand, when the samples from different states do not have any overlaps in the configuration space, ${\widehat{f}}_{i}$ in Eq. 3 fail to converge, or ${\widehat{f}}_{i}$ could have large uncertainties even after convergence. The uncertainties can be estimated by the analytical equation (given in (Shirts and Chodera 2008)) or by using the bootstrap method or the block averaging method. When the hidden energy barriers exist in a full dimensional space, the uncertainty in ${\widehat{f}}_{i}$ in the MBAR equation cannot decrease rapidly even with extended MD simulation trajectory data. To overcome this, better conformational sampling schemes applicable to higher dimensional spaces are necessary.

Once ${\widehat{f}}_{i}$ is obtained, thermodynamic averages in the unsampled state can be calculated by “extrapolating” or “interpolating” the MBAR equations from the samples obtained in simulations with *K* states. Let ${u}_{\mathrm{target}}({\varvec{x}})$ be the reduced potential energy in the target state where the average ${\langle A\rangle }_{\mathrm{target}}$ of the physical quantity $A({\varvec{x}})$ will be estimated. ${\langle A\rangle }_{\mathrm{target}}$ can be derived as follows:

4 $${\langle A\rangle }_{\mathrm{target}}=\frac{\int A\left({\varvec{x}}\right){e}^{-{u}_{\mathrm{target}}({\varvec{x}})}dx}{\int {e}^{-{u}_{\mathrm{target}}({\varvec{x}})}dx}=\frac{\mathrm{exp}\left[-{f}_{\mathrm{target},A}\right]}{\mathrm{exp}\left[-{f}_{\mathrm{target}}\right]}$$

Here, ${f}_{\mathrm{target},A}$ uses a non-Boltzmann weight because of the factor $A\left({\varvec{x}}\right)$. The estimates of the free energies, ${f}_{\mathrm{target},A}$, ${f}_{\mathrm{target}}$, ${\widehat{f}}_{\text{target },A}$, and${\widehat{f}}_{\text{target}}$, can be obtained by solving the above MBAR equations by regarding the ensembles as (*K* + 1)-th and (*K* + 2)-th states with ${N}_{K+1}=0$ and ${N}_{K+2}=0$, respectively:

5 $${\widehat{f}}_{\text{target }}=-\mathrm{ln}\sum_{j=1}^{K} \sum_{n=1}^{{N}_{j}} \frac{\mathrm{exp}\left[-{u}_{\text{target }}\left({{\varvec{x}}}_{jn}\right)\right]}{\sum_{k=1}^{K} {N}_{k}\mathrm{exp}\left[{\widehat{f}}_{k}-{u}_{k}\left({{\varvec{x}}}_{jn}\right)\right]}$$

6 $${\widehat{f}}_{\text{target },A}=-\mathrm{ln}\sum_{j=1}^{K} \sum_{n=1}^{{N}_{j}} \frac{A\left({{\varvec{x}}}_{jn}\right)\mathrm{exp}\left[-{u}_{\text{target }}\left({{\varvec{x}}}_{jn}\right)\right]}{\sum_{k=1}^{K} {N}_{k}\mathrm{exp}\left[{\widehat{f}}_{k}-{u}_{k}\left({{\varvec{x}}}_{jn}\right)\right]}$$

When the delta function (or indicator function) on order parameter(s) (or collective variable(s)) is used as the physical quantity $A({\varvec{x}})$, then the PMF on those coordinate(s) can be obtained.

**Fig. 1:**
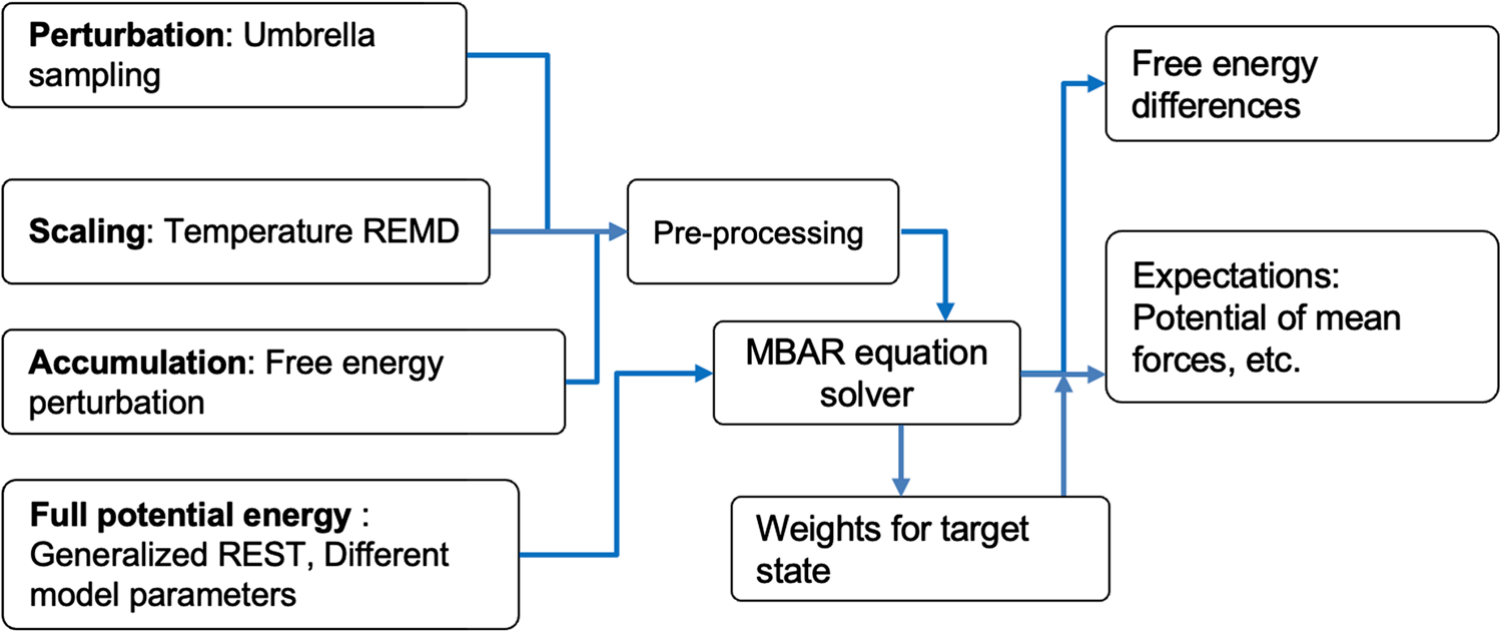
Classifications of four major applications for the MBAR equations and the schemes from input data to output results

In principle, the MBAR equations can utilize the potential energy ${U}_{i}({\varvec{x}})$, volume $V\left({\varvec{x}}\right)$, and other thermodynamic data as input data. However, evaluating the whole system’s potential energies for all combinations at different states is a time-consuming process. Obviously, some applications can simplify or skip this process. For this purpose, we categorize four major usages of the MBAR equations: (i) perturbation, (ii) scaling, (iii) accumulation, and (iv) full potential energy as shown in Fig. 1. In the following, we describe the reduced MBAR equations and input data for each case.

### Perturbation: umbrella sampling

Here, we mean that “perturbation” is to add an additional term to the original potential energy that is used in MD simulations. Umbrella sampling (Torrie and Valleau 1977) imposes a restraint force(s) along collective variable(s) ${\varvec{z}}({\varvec{x}})$ and is considered as a major example of “perturbation.” The potential energy used in umbrella sampling can be decomposed to ${U}_{i}\left(x\right)={U}_{\mathrm{system}}\left({\varvec{x}}\right)+{U}_{restraint}^{\left(i\right)}\left({\varvec{z}}({\varvec{x}})\right)$. Here, ${U}_{\mathrm{system}}\left({\varvec{x}}\right)$ is the potential energy from the system’s force-field shared at all the umbrella windows, and ${U}_{restraint}^{\left(i\right)}\left({\varvec{z}}({\varvec{x}})\right)$ is a restraint energy for the *i*-th umbrella window. The quadratic form ${k}_{i}{\left({\varvec{z}}({\varvec{x}})-{{\varvec{z}}}_{i}^{\mathrm{ref}}\right)}^{2}$ is usually used for ${U}_{restraint}^{\left(i\right)}\left({\varvec{z}}({\varvec{x}})\right)$. In the context of umbrella sampling, states *i* in the MBAR analysis are specified by ${U}_{restraint}^{\left(i\right)}\left({\varvec{z}}({\varvec{x}})\right)$. When ${U}_{i}\left({\varvec{x}}\right)$ is used in the MBAR equations, the terms of ${U}_{\mathrm{system}}\left({\varvec{x}}\right)$ are canceled out, so only ${U}_{restraint}^{\left(i\right)}\left({\varvec{z}}({\varvec{x}})\right)$ is needed as input. To simplify further, only ${k}_{i}$, ${{\varvec{z}}}_{i}^{\mathrm{ref}}$, and ${\varvec{z}}({{\varvec{x}}}_{jn})$ are needed as the input data. The resulting MBAR equations for the (dimensionless) free energies of umbrella window systems are:

7 $${\widehat{f}}_{i}=-\mathrm{ln}\sum_{j=1}^{K} \sum_{n=1}^{{N}_{j}} \frac{\mathrm{exp}\left[-\beta {U}_{restraint}^{\left(i\right)}\left({\varvec{z}}({{\varvec{x}}}_{jn})\right)\right]}{\sum_{k=1}^{K} {N}_{k}\mathrm{exp}\left[{\widehat{f}}_{k}-\beta {U}_{restraint}^{\left(k\right)}\left({\varvec{z}}({{\varvec{x}}}_{jn})\right)\right]}=-\mathrm{ln}\sum_{j=1}^{K} \sum_{n=1}^{{N}_{j}} \frac{\mathrm{exp}\left[-\beta {k}_{i}{\left({\varvec{z}}({{\varvec{x}}}_{jn})-{{\varvec{z}}}_{i}^{\mathrm{ref}}\right)}^{2}\right]}{\sum_{k=1}^{K} {N}_{k}\mathrm{exp}\left[{\widehat{f}}_{k}-{\beta k}_{k}{\left({\varvec{z}}({{\varvec{x}}}_{jn})-{{\varvec{z}}}_{k}^{\mathrm{ref}}\right)}^{2}\right]}$$

Typically, umbrella sampling aims to obtain the PMF force along ${\varvec{z}}$ under the ensemble of the target state as the restraint-free state. In this case, ${u}_{\text{target }}\left({\varvec{x}}\right)=0$ is used as the reduced energy, which can be calculated without further input data.

### Scaling: temperature replica exchange MD

In “scaling,” a scaling of the original potential energy by a factor is applied in MD simulations. We here consider T-REMD as an example of “scaling.” In the context of T-REMD, states *i* in the MBAR analysis are specified by ${\beta }_{i}$. The reduced potential energy used in T-REMD is ${u}_{i}\left(x\right)={\beta }_{i}{U}_{\mathrm{system}}\left({\varvec{x}}\right)$, just a scaling of the original potential energy ${U}_{\mathrm{system}}\left({\varvec{x}}\right)$. In this case, the resulting MBAR equation is:

8 $${\widehat{f}}_{i}=-\mathrm{ln}\sum_{j=1}^{K} \sum_{n=1}^{{N}_{j}} \frac{\mathrm{exp}\left[-{\beta }_{i}{U}_{\mathrm{system}}\left({{\varvec{x}}}_{jn}\right)\right]}{\sum_{k=1}^{K} {N}_{k}\mathrm{exp}\left[{\widehat{f}}_{k}-{\beta }_{k}{U}_{\mathrm{system}}\left({{\varvec{x}}}_{jn}\right)\right]}$$

Here, ${\beta }_{i}{U}_{\mathrm{system}}\left({{\varvec{x}}}_{jn}\right)$ can be calculated by multiplying ${U}_{\mathrm{system}}\left({{\varvec{x}}}_{jn}\right)$ by ${\beta }_{i}$. Therefore, the only inputs required for the MBAR equations are the set of ${U}_{\mathrm{system}}\left({{\varvec{x}}}_{jn}\right)$ and ${\beta }_{i}$. For the calculation of thermodynamic averages or PMF, the target ensemble, i.e., ${u}_{\mathrm{target}}\left({\varvec{x}}\right)={\beta }_{1}{U}_{\mathrm{system}}\left({{\varvec{x}}}_{jn}\right)$, at the lowest temperature, ${\beta }_{1}$. Note that making use of different temperatures like Eq. (8) using the WHAM instead of the MBAR requires an advanced reformulation of the WHAM theory, such as the parallel tempering WHAM (Chodera et al. 2007). Here, we assume the NVT ensemble, but in the NPT ensemble, we must also consider $pV$ as the inputs (Paschek and García 2004) (Peter et al. 2016).

### Accumulation: free-energy perturbation

“Accumulation” is defined as calculations that are accumulating or integrating the free energy difference between states. As a typical example of “accumulation,” we here consider free-energy perturbation (FEP) (Zwanzig 1954) (Tembre and McCammon﻿ 1984) (Mey et al. 2020), which is typically used to calculate the free energy difference in alchemical transformation. In FEP, multiple intermediate states are stratified with a one-dimensional control parameter λ ($0\le \lambda \le 1$). By stratifying the start ($\lambda =0$) and end ($\lambda =1$) states, the distributions of reduced energies of neighboring states over *λ* can overlap with each other, resulting in more accurate estimates of the free-energy differences between neighboring states, $\Delta{f}_{i,i+1}$. Theoretically, the free-energy difference between the start and end states is obtained by accumulating the neighboring differences, $\Delta{f}_{\mathrm{start},\mathrm{ end}}=\sum \Delta{f}_{i,i+1}$. In FEP, the potential energy is described by $U\left(x;{\lambda }_{i}\right)=\left(1-\lambda \right){U}_{\mathrm{start}}\left(x\right)+\lambda {U}_{\mathrm{end}}(x)$. In the context of FEP, states *i* in the MBAR analysis are specified by $U\left(x;{\lambda }_{i}\right)$. In the MBAR analysis of FEP data, it is ideal to prepare the reduced energies for all combinations of stratified states ${u}_{i}\left({{\varvec{x}}}_{jn}\right)$, and to solve the full MBAR equations. However, for practical uses, it is often sufficient for keeping accuracy of the free-energy difference to care about the reduced energies only between neighboring states. It is thus not worth the cost to calculate the reduced energies for all combinations of the states (Paliwal and Shirts 2011). Also, the difference of the reduced energies between non-neighboring states often becomes too large, leading to the instability of energy calculations. In the case of scaling Lennard–Jones (LJ) interactions of a solute in water, the solute can overlap with water molecules in the end state ($\lambda =0$, i.e., the fully decoupled state of the solute). The reduced energy of the start state ($\lambda =1$; the fully coupled state of the solute) estimated using the configuration of the end state becomes infinite even though the soft-core modification (Beutler et al. 1994) (Zacharias et al. 1994, p.) is introduced into the LJ interactions.

In our framework, only the reduced energies of the neighboring states, i.e., ${u}_{i}\left({{\varvec{x}}}_{in}\right)$, ${u}_{i+1}\left({{\varvec{x}}}_{in}\right)$, are used as input data. The free-energy differences between the neighboring states are accumulated to obtain the difference between the start and end states. In this sense, MBAR for “perturbation” is essentially the same framework as the original BAR.

### Full potential energy: REST, parameter tuning, and others

The last case, “full potential energy,” needs the whole system’s potential energies for solving the MBAR equations. In this context, state *i* in the MBAR analysis is specified by the whole systems’ potential energy. In replica exchange with solute tempering (REST) (Liu et al. 2005), REST2 (Wang et al. 2011) (Terakawa et al. 2011), and gREST (Kamiya and Sugita 2018), solute molecules, such as proteins or ligands (in REST/REST2), or part of solute molecules with full or partial potential energies (in gREST) are scaled as a solute region. In REST/REST2/gREST simulations, the solute region in each replica has a different temperature, while the temperatures in the solvent region in all replicas are the same, for instance, room temperature. Although only the scaled terms can be given to the MBAR equations ignoring the other canceling terms, most MD software does not write specific terms of the potential energy by default, but evaluates potential energies from updated parameters of the solute region. Thus, in terms of computational cost, there is no difference in preparing the potential energy for the entire system or the specific scaled terms. Another example is the tuning of force field parameters or model parameters (Messerly et al. 2018)(Shinobu et al. 2019). Since parameters affect the total energy in a complicated way, we need to prepare the potential energies of all the replicas in the whole system. In the case of parameter tuning, parameter sets, which have not yet been sampled, are used as the target state of the MBAR for predicting the behavior of new parameter sets.

Further complicated yet important case is a combination of umbrella sampling (perturbed case) and different potential energy functions (case of full potential energy) in hybrid quantum mechanics/molecular mechanic (QM/MM) calculations (Warshel and Levitt 1976) (Yagi et al. 2021). In the MBAR analysis using the QM/MM data, it is possible to reweight samples obtained by low-level theory (LL, e.g., classical force field or semi-empirical QM) with the energies of high-level theory (HL, e.g., ab initio QM) and obtain more accurate PMF. Because the computational cost of MD simulations with HL is far greater than with that with LL, various methods have been proposed to correct LL data with HL calculations as post-processing (Yagi et al. 2021). LL calculation is often conducted with umbrella sampling along a reaction coordinate $z({\varvec{x}})$. Then, the total potential energy of the LL with umbrella window becomes ${U}_{i}\left(x\right)={U}_{\mathrm{system}}^{\mathrm{LL}}\left({\varvec{x}}\right)+{U}_{\mathrm{restraint}}\left(z({\varvec{x}})\right)$. The target potential energy is the potential energy of the HL system without restraint potential, ${u}_{\text{target }}\left({\varvec{x}}\right)={U}_{\mathrm{system}}^{\mathrm{HL}}\left({\varvec{x}}\right)$. The required input data are the potential energies of the LL ${U}_{\mathrm{system}}^{\mathrm{LL}}\left({{\varvec{x}}}_{n}\right)$ and HL ${U}_{\mathrm{system}}^{\mathrm{HL}}\left({{\varvec{x}}}_{n}\right)$, and the restraint energies ${U}_{\mathrm{restraint}}^{\left(i\right)}\left(z({{\varvec{x}}}_{n})\right)$. The resulting MBAR equations are:

9 $${\widehat{f}}_{i}=-\mathrm{ln}\sum_{j=1}^{K} \sum_{n=1}^{{N}_{j}} \frac{\mathrm{exp}\left[-{\beta }_{i}{U}_{\mathrm{system}}^{\mathrm{HL}}\left({{\varvec{x}}}_{jn}\right)\right]}{\sum_{k=1}^{K} {N}_{k}\mathrm{exp}\left[{\widehat{f}}_{k}-{\beta }_{k}\left\{{U}_{\mathrm{system}}^{\mathrm{LL}}\left({{\varvec{x}}}_{jn}\right)+{U}_{\mathrm{restraint}}^{\left(k\right)}\left({{\varvec{x}}}_{jn}\right)\right\}\right]}$$

### Implementation

Based on the above classifications, we implemented a MBAR code, which we call mbar_analysis in the GENESIS software package (Jung et al. 2015) (Kobayashi et al. 2017). The implemented code preprocesses the input data for each one of the above classifications, and the input formats are thus simplified as much as possible. The code calls a common MBAR equation solver to estimate free-energy differences. The solver was implemented by combining a simple self-consistent iteration with the Newton–Raphson method. The calculation of the denominator of the MBAR equation was parallelized with multi-threads, resulting in a faster execution than the reference MBAR implementation, PyMBAR (Shirts and Chodera 2008). After obtaining the estimates, it gives us the free-energy difference, weights, and PMF according to the input parameters. The code was implemented in FORTRAN. 

## Demonstration of MBAR analysis using mbar_analysis 

### Test simulation systems

We first demonstrate the MBAR analysis in the case of “perturbation” using alanine-tripeptide in vacuum as a target molecule. Using CHARMM36m force field (Huang et al. 2017), the umbrella sampling simulation was performed with GENESIS atdyn (Jung et al. 2015), (Kobayashi et al. 2017). The $\omega$ angle values were increased by 3 degrees between the centers of neighboring windows, resulting in 61 windows to investigate $\omega$ angle values from 0 to 180. A spring constant of 200 kcal/mol/rad^2^ was applied. Temperature was controlled at 300 K by the stochastic velocity scaling method (Bussi et al. 2007). Long-range electrostatic interactions were treated without cutoff. Instantaneous $\omega$ angles corresponding to $z({{\varvec{x}}}_{jn})$ in the MBAR equations were extracted from the trajectory data with trj_convert tool in GENESIS. Extracted angle values, the spring constants, and the window centers were used as the input of the MBAR. For reference, the same calculation was performed with the WHAM implemented as wham_analysis tool in GENESIS. 

To demonstrate the MBAR analysis of T-REMD data, we performed a T-REMD simulation of alanine-tripeptide in solution. The initial structures and parameters are the same as those used in GENESIS tutorials (https://www.r-ccs.riken.jp/labs/cbrt/tutorials2022/). We calculated the potential of mean force (PMF) in the $\phi$ and $\psi$ dihedral angle space. Using CHARMM36 force field (Huang and MacKerell 2013) and TIP3P water molecules (Jorgensen et al. 1983), the simulation was performed with GENESIS spdyn. In MD, electrostatic interactions were treated by the smooth Particle Mesh Ewald (Essmann et al. 1995), and covalent bonds containing hydrogen atoms were constrained by the SHAKE (Ryckaert et al. 1977) or SETTLE (Miyamoto and Kollman 1992) algorithms. The temperature was controlled by the stochastic velocity scaling method (Bussi et al. 2007). These methods were kept in other applications. The system’s potential energy was extracted from the MD log file and sorted from replica-ID data to temperature-ID data by using remd_convert tool in GENESIS. Then, they were used as the input together with the temperatures of the replicas for the MBAR. Also, the dihedral angles $\phi$ and $\psi$ were extracted (with trj_convert tool) from the trajectory data and used as the input to calculate PMF at 300 K. We compared PMF using the trajectory only at 300 K with that of all temperature trajectories reweighted using MBAR. 

For a demonstration of the “accumulation” case, we calculated a mutation from the amino-acid side-chain analogue of Val to that of Trp in water. Twenty strata were used to divide $\lambda$ into equal intervals from $\lambda =0$ to $\lambda =1$, and FEP was performed. Using CHARMM36 force field and TIP3P water model, the simulation was performed with the FEP implementation (Oshima et al. 2020) in GENESIS spdyn. To remove the instability of MD simulations near end states, the soft-core treatment is introduced to the LJ and electrostatic potentials (Zacharias et al. 1994) (Steinbrecher et al. 2011). The reduced energies of the neighboring states, i.e., ${u}_{i-1}\left({{\varvec{x}}}_{in}\right)$, ${u}_{i}\left({{\varvec{x}}}_{in}\right)$, ${u}_{i+1}\left({{\varvec{x}}}_{in}\right)$ were generated with GENESIS spdyn, and they were used as the input for the MBAR analysis. 

As a demonstration of a case of “full potential energy,” gREST of alanine-tripeptide in water (taken from GENESIS tutorials) was performed using four replicas of alanine-tripeptide as solute and temperature control by scaling all potential energy terms. Using CHARMM36 force field and TIP3P water model, the simulation was performed with GENESIS spdyn. The potential energies required in MBAR were generated using the optional function of GENESIS spdyn. The potential energy data of replicas were sorted from replica-ID data to temperature-ID data using remd_convert tool of GENESIS. We calculated PMF along the distance between the terminal residues (an oxygen of the ALA1 and a hydrogen of ALA3). We compared PMF using the trajectory at 300 K with that of all replicas’ trajectories reweighted by the MBAR.

Finally, as a demonstration of QM/MM with umbrella sampling MD simulation (QM/MM-US-MD), QM/MM-US-MD of malonaldehyde (MA) and p-Nitrophenyl phosphate (pNPP^2−^) were performed at the third-order extension Self-Consistent-Charge Density Functional Tight-Binding level (Gaus et al. 2011, p. 3). MA and pNPP^2−^ are encapsulated in a TP3P water sphere with a radius of 20 Å. The reaction coordinate for MA is a linear combination of two distances between atoms involved in the proton transfer reaction. The reaction coordinate for pNPP^2−^ is the P-O distance involved in hydrolysis (Fig. 3a and b).

For MA, MD calculations were performed for 200 ps at 300 K using a spring constant of 40 kcal/mol/rad^2^ in 21 umbrella windows centered along the reaction coordinate increased from − 1.0 to 1.0 at 0.1 Å intervals. For pNPP^2−^, MD calculations were performed for 500 ps at 300 K using a spring constant of 300 kcal/mol/rad^2^ in 18 umbrella windows centered along the reaction coordinate increased from − 1.4 to 3.1 at 0.1 Å intervals. The 3ob (ophyd) parameter (Gaus et al. 2013)(Gaus et al. 2014) was used as the Slater-Koster parameter, and CHARMM36m (Huang et al. 2017) and CGenFF (Vanommeslaeghe et al. 2009) (Yu et al. 2012) were used as the classical force fields. For reweighting with MBAR, potential energies were evaluated by single-point energy calculation for 2000 and 5000 samples for MA and pNPP2- at the density functional theory (DFT) level using B3LYP/cc-pvdz (Lee et al. 1988) (Becke 1993) (Grimme et al. 2010) (Dunning 1989), respectively. DFTB and DFT calculations were performed using QSimulate (https://qsimulate.com), a fast quantum computation program package, in combination with GENESIS atdyn (Yagi et al. 2021). 

### Demonstrations of mbar_analysis

**Fig. 2:**
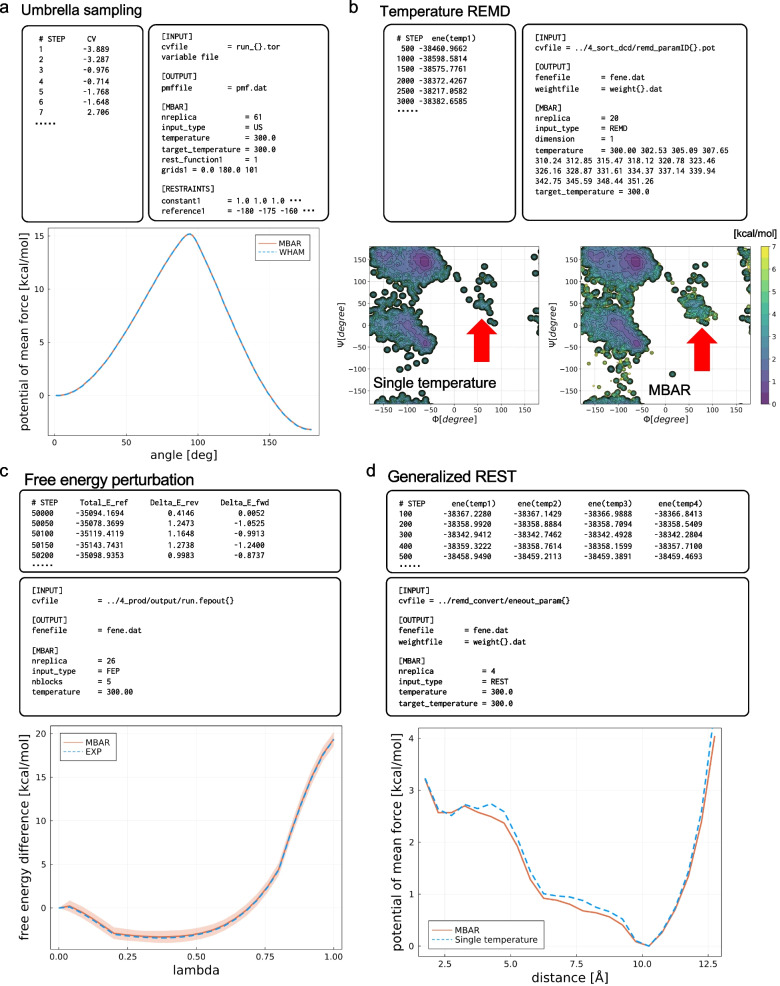
Results of the MBAR analysis for four cases of our classification. **a** Potential of mean force (PMF) along $\omega$ dihedral angle of alanine-tripeptide in vacuum. The results of MBAR and WHAM are indicated by red solid line and blue dashed lines, respectively. **b** PMF in $\phi$ and $\psi$ dihedral angles of alanine-tripeptide in water. The PMF calculated only from the trajectory of 300 K and all trajectories reweighted by the MBAR are shown. The region of the left-handed helix is indicted by red arrow. **c** Free-energy differences upon the mutation from the amino-acid side-chain analogue of Val to that of Trp as a function of $\lambda$, calculated by free-energy perturbation method. The shaded region indicates the uncertainties estimated by block averaging. EXP means the exponential averaging. **d** PMF along the distance between the terminal residues of alanine-tripeptide in water, calculated by gREST simulation. The PMF calculated only from the trajectory of 300 K and all trajectories reweighted by the MBAR are shown

Figure 2A shows the inputs required for the MBAR analysis and the results of the umbrella sampling of alanine-tripeptide in vacuum. As explained above, the only inputs required for umbrella sampling are the collective variable trajectories of umbrellas, ${\varvec{z}}({{\varvec{x}}}_{jn})$, the window centers, and the spring constants. Restraint energies, ${U}_{\mathrm{restraint},i}\left({{\varvec{x}}}_{jn}\right)$, required in the MBAR equations, are calculated internally and passed to the solver. In the PMF calculation with WHAM, the density of states is calculated after making the histogram of the trajectories, whereas in the MBAR, the density of states for each ${\varvec{z}}$ is calculated after the weight of each sample point. Therefore, theoretically, MBAR can prevent any biases due to convolutions.

Figure 2b shows the inputs required for the subsequent MBAR analysis and the results of the T-REMD of alanine-tripeptide in water. As explained above, the only inputs required for T-REMD are the potential energies of sorted replicas and temperatures. The reduced energies ${\beta }_{i}{U}_{\mathrm{system}}\left({{\varvec{x}}}_{jn}\right)$, required in the MBAR equations, are calculated internally and passed to the solver. Comparing the PMF obtained from only the lowest temperature 300 K trajectory with that weighted all replica trajectories with MBAR, the left-handed helix state is well captured in MBAR. This is because the left-handed helix sampled at high temperature is not discarded but treated as the weighted samples by the MBAR.

Figure 2c shows the inputs required in the subsequent MBAR analysis and the results of the alchemical FEP from Val to Trp in water. As explained above, the only input required for FEP is the potential energies of the neighboring states. Our MBAR tool integrates the free-energy difference between the neighboring states to obtain the total difference between the start and end states. Inside the program, the MBAR equation for two states is called repeatedly. The free energy difference between Val and Trp shown in Fig. 2c is comparable with the exponential averaging, and matched with the result obtained with NAMD (Liu et al. 2012), indicating that the potential energy difference from the distant state is not contributing. This is also consistent with the result of the exhaustive FEP benchmarks by Paliwal and Shirts (2011), where they showed that MBAR and BAR have comparative accuracies for many FEP data analyses.

Figure 2d shows the results of gREST for alanine-tripeptide in water and the inputs required in the MBAR analysis. The reduced energies required for MBAR were generated with GENESIS spdyn during the simulations. As shown in the figure, the potential energies with all scaling values are required as input for the MBAR analysis. Figure 2d compares the PMF at the lowest temperature (i.e., scaling factor) trajectory with that using MBAR. The figure shows that the PMF using MBAR well captures the stable conformations indicated at the lower PMF values compared to the PMF values using a single trajectory at 300 K. 

**Fig. 3:**
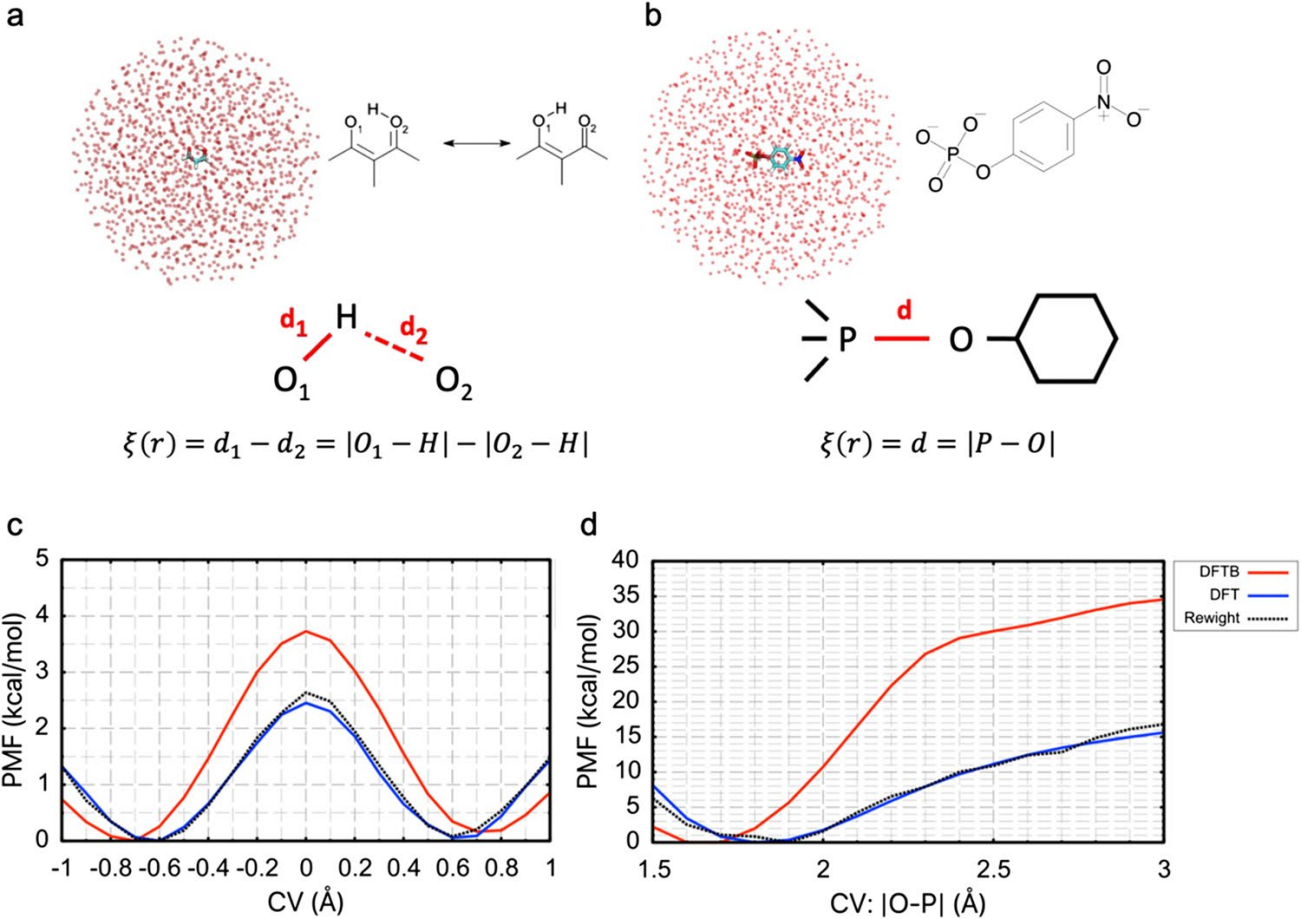
Simulation system and result of QM/MM with umbrella sampling simulations. **a** The malonaldehyde is located at the center of the water droplet. **b** The pNPP^2−^ is located at the center of the water droplet. The collective variables used in the umbrella sampling are indicted in the figure. **c** The potential of mean force (PMF) of the malonaldehyde obtained by DFTB (red line), DFT (blue line), and re-weighted with the MBAR (black dotted line). **d** PMF of the pNPP^2−^

Figure 3c and d shows the PMF obtained in DFTB, DFT, and reweighted PMF with MBAR. A total of 10 ps (10,000 MD steps) equilibration followed by 20 ps (20,000 MD steps) US-MD calculations were performed in MA and pNPP^2−^ to obtain PMF at the DFT level. In both systems, the PMF obtained from the DFT-level calculation and the PMF obtained from the reweighting are in good agreement. The computational cost for reweighting was 6.7% for MA (16.7% for pNPP^2−^) of the 30,000 MD steps of the brute-force DFT-US-MD calculation (only 2000 samples were reweighted for MA, and 5000 samples were reweighted for pNPP^2−^). Furthermore, as only the potential energy is required for reweighting, the gradient calculation can be omitted, further reducing the actual computational cost.

## Discussion

MBAR is a very flexible method, which can handle various states, ranging from different temperature/pressure conditions to different force field parameters. On the other hand, this flexibility sometimes requires complicated inputs, even for straightforward analyses such as umbrella sampling. In this review, we classified typical applications of MBAR and simplifying input data for practical usages. Our classification would help non-expert users to apply the MBAR analysis to obtain unbiased thermodynamic data from biased MD simulation trajectories.

One important future application of MBAR would be feedback to simulation models, as shown by Messerly et al. (2018) and Shinobu et al. (2019). Since MBAR extrapolates and estimates statistics on parameter sets that have not yet been sampled, it helps us to optimize the parameters of the simulation model. When the number of parameters to be optimized is large, grid search becomes difficult even with the help of the MBAR equations. In this case, as recently shown by Wieder et al. (2021), by making the outputs of the MBAR equations differentiable with respect to potential energies and their force field parameters, efficient gradient search can be performed. It is actually similar to the training of neural network model parameters. On the other hand, the limitation of MBAR in terms of feedback to the simulation model is that the MBAR is just an estimator; thus, it can only estimate free-energy differences or the averaged data. Recent deep learning technologies are also flexible, and they can directly use modeling of the conformational density of molecules as well as statistical averages. For example, Wang et al. recently succeeded in modeling conformational density from various temperature trajectories obtained by T-REMD using a diffusion model in which temperature is incorporated as one of the random variables (Wang et al. 2022). If it becomes possible to model the density and the uncertainty of parameters and conformations with these technologies, more efficient optimization will be possible in combination with MBAR.

## Data Availability

The developed code has been released to the public as one of the analysis tools (called mbar_analysis) of MD simulation software GENESIS (Jung et al. 2015) (Kobayashi et al. 2017). This software is freely available from https://www.r-ccs.riken.jp/labs/cbrt/. The inputs used in the calculations in this review are available at https://github.com/matsunagalab/paper_mbar, except for the inputs for the QM/MM simulations, which are available from the authors upon reasonable request.
